# TNFAIP3 interacting protein 2 relieves lipopolysaccharide (LPS)‐induced inflammatory injury in endometritis by inhibiting NF‐kappaB activation

**DOI:** 10.1002/iid3.970

**Published:** 2023-10-13

**Authors:** Xinxin Qian, Yan Wang, Xingmei Li, Yuewen Li, Liping Li

**Affiliations:** ^1^ Department of Gynecology The Third Affiliated Hospital of Qiqihar Medical College Qiqihar China

**Keywords:** endometritis, inflammation, NF‐κB, oxidative stress, TNIP2

## Abstract

**Background:**

Endometritis seriously affects the health of women, and it is important to identify new targets for its treatment.

**Objective:**

This study aimed to explore the role of TNFAIP3 interacting protein 2 (TNIP2) in endometritis through human endometrial epithelial cells (hEECs) stimulated by lipopolysaccharide (LPS).

**Methods:**

hEECs were induced with LPS to build a cellular model of endometritis. Cell growth and apoptosis were detected by cell counting kit‐8 and flow cytometry. The TNIP2 mRNA and protein levels were measured using reverse transcription quantitative polymerase chain reaction (RT‐qPCR) and western blot analysis, respectively. The caspase3 activity was calculated using a Caspase3 activity kit. Interleukin (IL)−1β, IL‐6, and tumor necrosis factor‐alpha (TNF‐α) levels were determined by enzyme‐linked‐immunosorbent‐assay. The reactive oxygen species (ROS), lactate dehydrogenase (LDH), catalase (CAT), and superoxide dismutase (SOD) levels were determined using the corresponding kits. Nuclear factor‐kappaB (NF‐κB) pathway was determined by western blot assay.

**Results:**

TNIP2 was downregulated in the LPS‐induced endometritis cell model. Cell viability was reduced, apoptosis was enhanced, and IL‐6, IL‐1β, and TNF‐α levels increased in LPS‐induced hEECs. Additionally, LDH activity and ROS concentration were upregulated, whereas CAT and SOD activities were downregulated in LPS‐induced hEECs. These results were reversed by TNIP2 overexpression. Moreover, the results hinted that NF‐κB was involved in the effects of TNIP2 on the LPS‐induced endometritis cell model.

**Conclusion:**

TNIP2 alleviated endometritis by inhibiting the NF‐κB pathway, suggesting a potential therapeutic target for endometritis.

## Graphical abstract

The present study explored the role of TNFAIP3 interacting protein 2 (TNIP2) in endometritis that stimulated by lipopolysaccharide (LPS) at human endometrial epithelial cells (hEECs). The findings revealed that TNIP2 alleviated endometritis by inhibiting the NF‐kB pathway, suggesting a potential therapeutic target for endometritis.

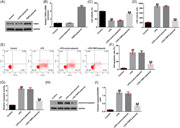

## 1 INTRODUCTION

Endometritis, a common gynecological disease, is an inflammatory transformation of the endometrial structure caused by various factors.^1^ Irregular menstruation, increased leukorrhea, pelvic pain, and dysmenorrhea are common symptoms. Without effective control, it may cause uterine myositis.^2^ If treatment is incomplete, repeated flare‐ups of inflammation will occur. Clinically, antibiotics and artificial estrogen and progesterone cycles are often used as treatment.^3^, ^4^ However, the clinical efficacy of this method is disappointing; thus, there is an urgent need to identify a new biomarker for endometritis.

TNFAIP3 interacting protein 2 (TNIP2) was a protein‐coding gene that encoded a protein identified as a nuclear factor‐kappaB (NF‐κB) activation suppressor.^5^ TNIP2 is involved in many disease processes. Chiang et al. indicated that TNIP2 mediates depressive disorder via GRβ.^6^ TNIP2 is also associated with pulmonary arterial hypertension.^7^ Feng et al. suggested that TNIP2 could alter lung cancer resistance to crizotinib.^8^ In female morbidity, TNIP2 is influenced by miR‐423 to promote breast cancer development.^9^ However, it remains unclear whether TNIP2 affects endometritis. Therefore, this study aimed to determine the effect of TNIP2 on endometritis.

The NF‐κB signaling pathway is involved in regulating apoptosis, glucose metabolism, and proliferation.^10^ Recently, it has been reported that an abnormal NF‐κB pathway may result in various human diseases, such as Parkinson's disease,^11^ cancers,^12^, ^13^ and type II diabetes mellitus.^14^ Moreover, recent studies revealed that the NF‐kB pathway was involved in *Staphylococcus aureus*‐induced mouse endometrial inflammation.^15^ Nevertheless, whether TNIP2 mediated the endometritis through the NF‐κB pathway is still unknown.

Lipopolysaccharide (LPS) is a major component of the cell wall of gram‐negative bacteria and can activate inflammatory cells.^16^ Human endometrial epithelial cells (hEECs) treatment with LPS have been widely used to study endometritis in vitro.^17^, ^18^, ^19^

Therefore, we conducted this study to explore the effect of TNIP2 on endometritis induced by LPS and to elucidate its mechanism of action.

## 2 MATERIALS AND METHODS

### 2.1 Cell culture

We brought hEECs from the Procell Life Science & Technology Co., Ltd). The cells were cultured in Ham's F‐12 medium (Biological Industries, Kibbutz Beit Haemek) contained with 1% penicillin and streptomycin (Beyotime) and 12% fetal bovine serum (Gibco) in an atmosphere of 5% CO_2_, at 37°C.

The hEECs were treated with 1 μg/mL LPS (L5293, Merck) for 3 h to establish an endometritis model in vitro.^19^

### 2.2 Cell treatment

To over‐express TNIP2 in hEECs, control‐plasmid or TNIP2‐plasmid was transfected into hEECs using Lipofectamine 2000 (Invitrogen) for 24 h referring to the manufacturer's protocol.

The hEECs were transfected with control‐plasmid or TNIP2‐plasmid in the presence or absence of 50 μM CU‐T12‐9 (NF‐κB agonist, HY‐110353, purity: 98.94%, MedChemExpress) for 24 h, followed by treatment with 1 μg/mL LPS for 3 h. Then following experiments were performed. The concentration selection of the CU‐T12‐9 was based on the previously published study.^20^

### 2.3 Cell counting kit‐8 (CCK‐8) assay^21^

Cell growth was tested using a CCK‐8 kit (Fcmacs). After LPS stimulation, hEECs were resuspended and seeded into 96‐well plates with 2.5 × 10^3^ cells and treated with 10 μL detection solution for 1.5 h at 37°C with 5% CO_2_ in the dark. The optical density (OD) values were measured at 450 nm using an ultraviolet spectrophotometer (Infinite Pro, Tecan).

### 2.4 Cell apoptosis analysis^22^

A total of 2 × 10^5^ LPS‐induced cells were harvested with 0.5 × 10^3^ μL solution containing 5 μL Annexin V‐FITC and 5 μL propidium iodide (Beyotime) at room temperature in the dark for 35 min. The apoptotic rate was analyzed using flow cytometry (C6; Thermo Fisher Scientific) and the data were analyzed by Kaluza Analysis (version 2.1.1.20653; Beckman Coulter, Inc.).

### 2.5 Caspase3 activity assay^23^

Activity in caspase3 of cells was calculated using a caspase3 colorimetric kit (Beyotime). The culture medium and cells were then covered with trypsin (BI). Cell samples were incubated with lysis buffer (Proteintech) for 20 min. The samples were stored by centrifugation at 10,000 rpm for 10 min. Finally, the samples were analyzed using a microplate reader (Tecan).

### 2.6 Enzyme‐linked‐immunosorbent analysis (ELIZA)^24^

The cell culture supernatant was harvested and used for detecting the level of interleukin (IL)−1β, IL‐6, and tumor necrosis factor‐alpha (TNF‐α). The ELIZA kits were obtained from Beyotime Biotechnology. All procedures were performed per the manufacturer's instructions.

### 2.7 Western blot analysis^25^

hEECs lysis was performed using the RIPA buffer (Beyotime, Shanghai, China). Proteins were separated using 12% sodium dodecyl sulfate‐polyacrylamide gel electrophoresis and transferred to polyvinylidene fluoride membranes (Whatman). Phosphate buffered saline‐tween‐20 (PBST, Beyotime, Shanghai, China) and 5% nonfat milk powder (CST) were used to block the polyvinylidene fluoride (PVDF) membranes. The PVDF membrane was then incubated for 12 h with primary antibodies (Proteintech) against TNIP2 (15459‐1‐AP; Proteintech), Caspase3 (19677‐1‐AP; Proteintech), p65 NF‐κB (66535‐1‐Ig; Proteintech), and phospho‐p65 NF‐κB (ARG51518; Arigo). The next day, after blocking with a second antibody (Arigo), the pattern was visualized using an ECL luminescent solution (Sangon Biotech), and the grayscale value of the target was counted using ImageJ.

### 2.8 Lactate dehydrogenase (LDH) assay

The LDH levels of the cells were measured following co‐culture with LPS using an LDH activity kit (ARG81306; Arigo), following the manufacturer's instructions. The OD value of each well was quantified at 490 nm to analyze LDH activity.

### 2.9 Reactive oxygen species (ROS) assay

A ROS kit (ARG81192; Arigo) was used to measure the concentration of ROS after treatment with 1 g/mL LPS per the manufacturer's protocol. Absorbance was measured at 525 nm using a spectrophotometer (Thermo Fisher Scientific).

### 2.10 Measurement of the activities of catalase (CAT) and superoxide dismutase (SOD)

The CAT and SOD activities of cells were measured following co‐culture with LPS using CAT and SOD activity kits (Jiancheng) according to the manufacturer's protocol. The OD of each well was determined at 525 nm.

### 2.11 Reverse transcription quantitative polymerase chain reaction (RT‐qPCR) assay

Following the supplier's protocol, total RNA was recovered from cells using ISOLATION TRIzol buffer® (Multi Sciences), and cDNA was obtained from reverse‐transcribed RNA using an RT‐PCR kit (Yeasen). qPCR was performed using the PerfectStart® Sybr qPCR Mix (Vazyme). The expression levels of genes were calculated using the $2^{‐∆∆C_{t}}$ method.^26^ The primer sequences for TNIP2 and‐actin were as follows: β‐actin:5′‐CCATCGCCAGTTGCCGATCC‐3′ (F) and 5′‐GCGAGAGGAGCACAGATACCACCAA‐3′ (R); TNIP2:5′‐AAGTCCTGACCAGTCGGAACA‐3′ (F) and 5′‐CCAGCAGGGACGAATACGTG‐3′ (R).

### 2.12 Statistical analysis

All experiments were repeated for three times. Statistical analyses were performed using SPSS 19.0 statistical software (IBM Corp.). Data are presented as mean ± standard deviation from triplicate experiments. Differences between the two groups were assessed using Student's *t* test, whereas one‐way analysis of variance followed by Tukey's test was performed to analyze differences among multiple groups. Statistical significance was set at *p* < .05 means statistical difference.

## 3 RESULTS

### 3.1 TNIP2 expression was decreased in LPS‐induced endometritis cell model

**Figure 1:**
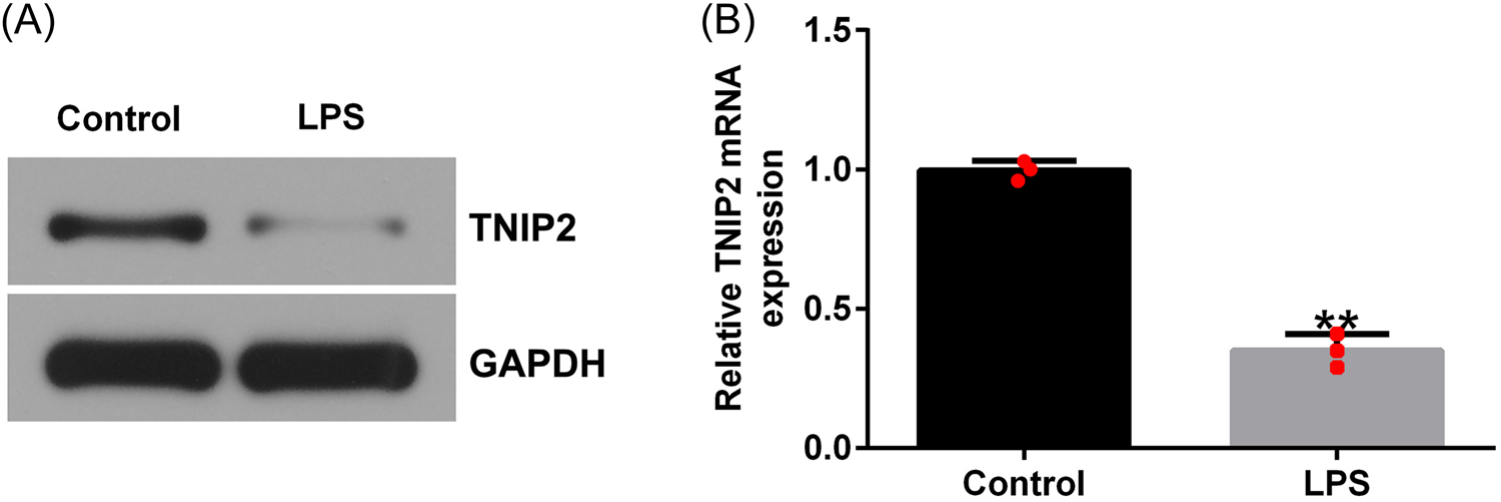
The TNIP2 expression level in LPS‐induced hEECs. (A) Western blot assay was performed to quantify the patterns of TNIP2. (B) The TNIP2 mRNA level in LPS‐induced hEECs. Data are exhibited as average ± SD of triple single experiments. *N* = 3. **p < .01 versus Control. hEECs, human endometrial epithelial cells; LPS, lipopolysaccharide; SD, standard deviation; TNIP2, TNFAIP3 interacting protein 2.

RT‐qPCR and western blot analysis were performed to determine TNIP2 expression in LPS‐induced endometritis cell model. Compared to the control group, the TNIP2 protein level was dramatically downregulated in the LPS‐stimulated group, and the results of RT‐qPCR were the same as those of western blot analysis (Figure 1A and B).

### 3.2 Overexpression of TNIP2 inhibited the apoptosis of hEECs

**Figure 2:**
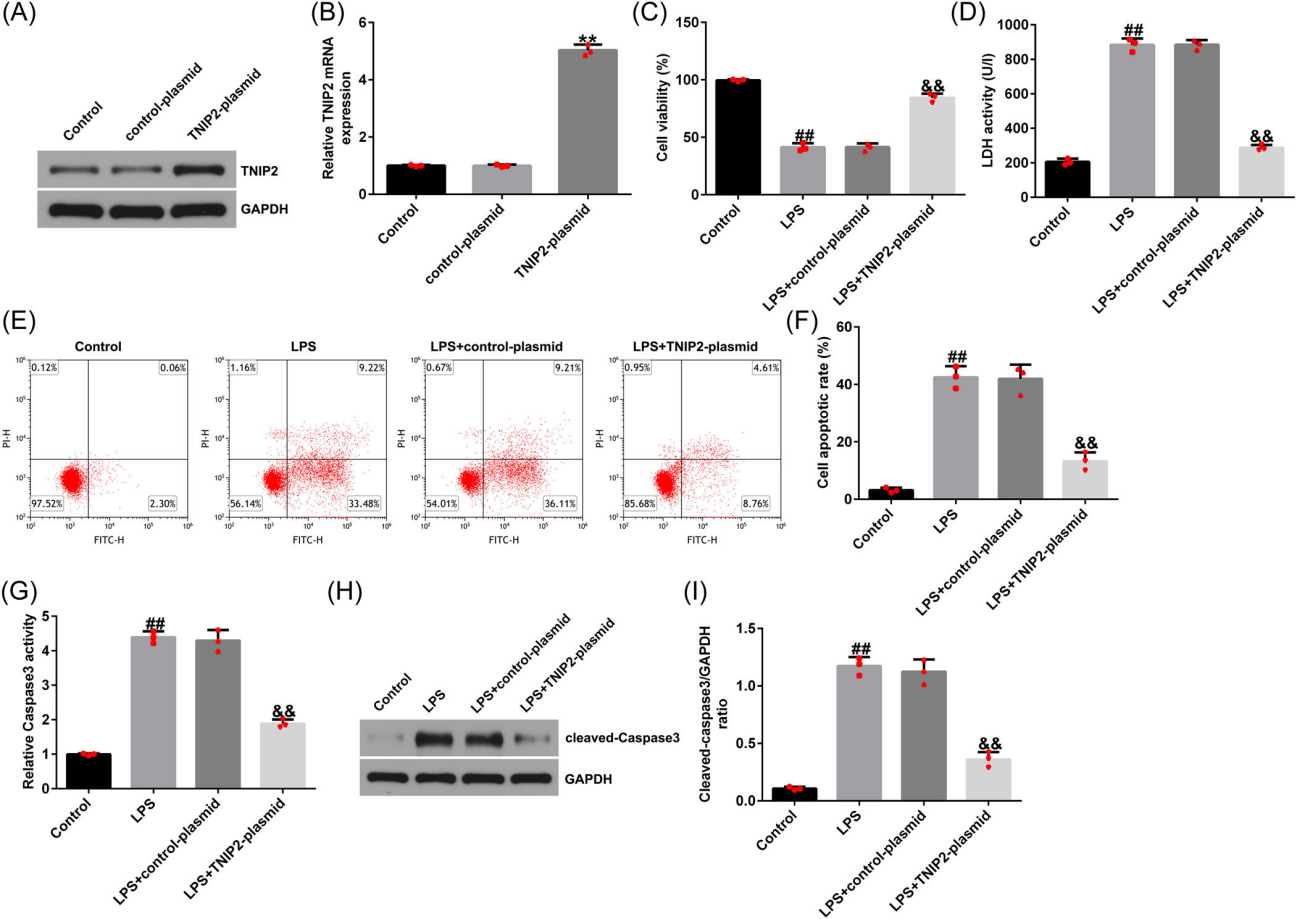
Effect of TNIP2 on apoptosis of LPS‐induced hEECs. (A) The efficiency of TNIP2‐plasmid transfection was determined by western blot assay. (B) The efficiency of TNIP2‐plasmid transfection was determined by RT‐qPCR. (C) CCK‐8 was carried out to calculate cell viability. (D) The LDH detection kit was used to measure LDH activity. (E and F) The FCM assay was carried out to assess cell apoptosis. (G) The capase3 activity detection kit was carried out to measure caspase3 activity. (H and I) Western blot analysis was performed to quantify the patterns of apoptosis‐related protein (cleaved‐caspase3). Data are exhibited as average ± SD of triple single experiments. *N* = 3. **p < .01 versus Control‐plasmid; ^##^ *p* < .01 versus Control; ^&&^ *p* < .01 versus LPS + control‐plasmid. CCK‐8, cell counting kit‐8; FCM, flow cytometry; hEECs, human endometrial epithelial cells; LDH, lactate dehydrogenase; LPS, lipopolysaccharide; SD, standard deviation; TNIP2, TNFAIP3 interacting protein 2.

To observe the effect of TNIP2 on the endometritis model, hEECs were pretransfected with control plasmid or TNIP2‐plasmid; 24 h later, the cells were treated with 1 μg/mL LPS for 3 h, and subsequent experiments were conducted. As shown in Figure 2A and B, the RT‐qPCR and western blot analysis results indicated that, compared to the control plasmid group, the TNIP2‐plasmid significantly increased the TNIP2 expression in hEECs. Compared to the control group, LPS treatment significantly inhibited cell viability (Figure 2C), enhanced the activity of LDH (Figure 2D), increased the rate of apoptosis (Figure 2E and F), increased the caspase3 activity (Figure 2G), and increased the cleaved‐caspase3 protein expression level in hEECs (Figure 2H and I). All of the above results were reversed by TNIP2‐plasmid transfection.

### 3.3 High TNIP2 expression inhibited the inflammation and oxidative stress in LPS‐induced hEECs

**Figure 3:**
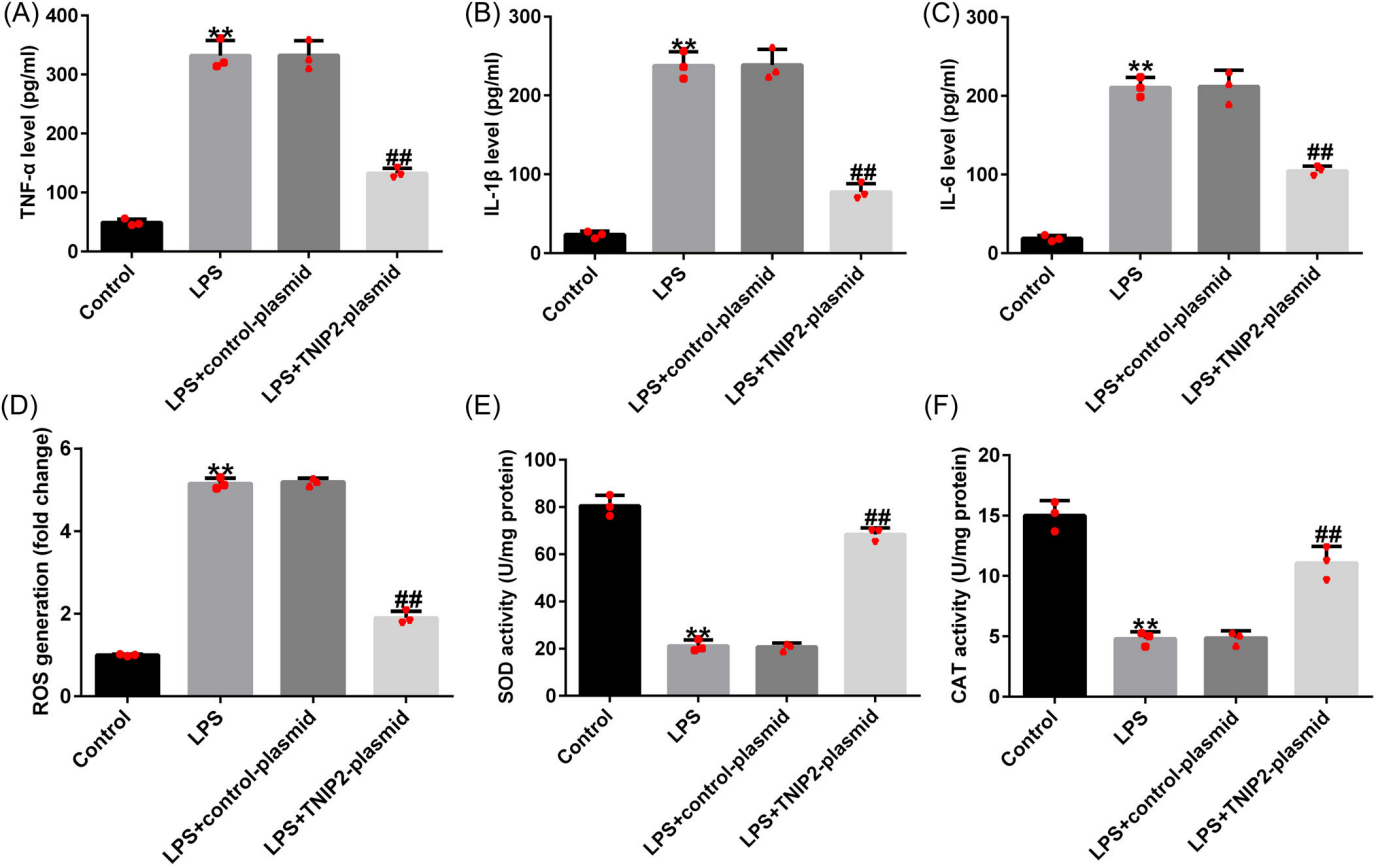
Impact of TNIP2 on inflammation and oxidative stress of LPS‐induced hEECs. (A–C) The concentration of TNF‐α, IL‐1β, and IL‐6 was detected by ELIZA. (D) ROS assay kit was performed to confirm the ROS release. (E and F) The SOD and CAT activity kits were enacted to detect the activity of SOD and CAT. Data are exhibited as average ± SD of triple single experiments. *N* = 3. **p < .01 versus Control; ^##^ *p* < .01 versus LPS + control‐plasmid. CAT, catalase; ELIZA, enzyme‐linked‐immunosorbent analysis; hEECs, human endometrial epithelial cells; IL, Interleukin; LPS, lipopolysaccharide; ROS, reactive oxygen species; SD, standard deviation; SOD, superoxide dismutase; TNF‐α, tumor necrosis factor‐alpha; TNIP2, TNFAIP3 interacting protein 2.

Endometritis is accompanied by the production of large quantities of inflammatory cytokines. Compared with the control group, the secretion of TNF‐α, IL‐1β, and IL‐6 in hEECs in the LPS group was significantly increased (Figure 3A–C). Similarly, released ROS levels were elevated in the LPS‐treated hEECs (Figure 3D). The activities of SOD and CAT were also disturbed (Figure 3E and F) in LPS‐induced hEECs. All of the above results were reversed by TNIP2‐plasmid transfection.

### 3.4 NF‐κB was activated in LPS‐induced hEECs

**Figure 4:**
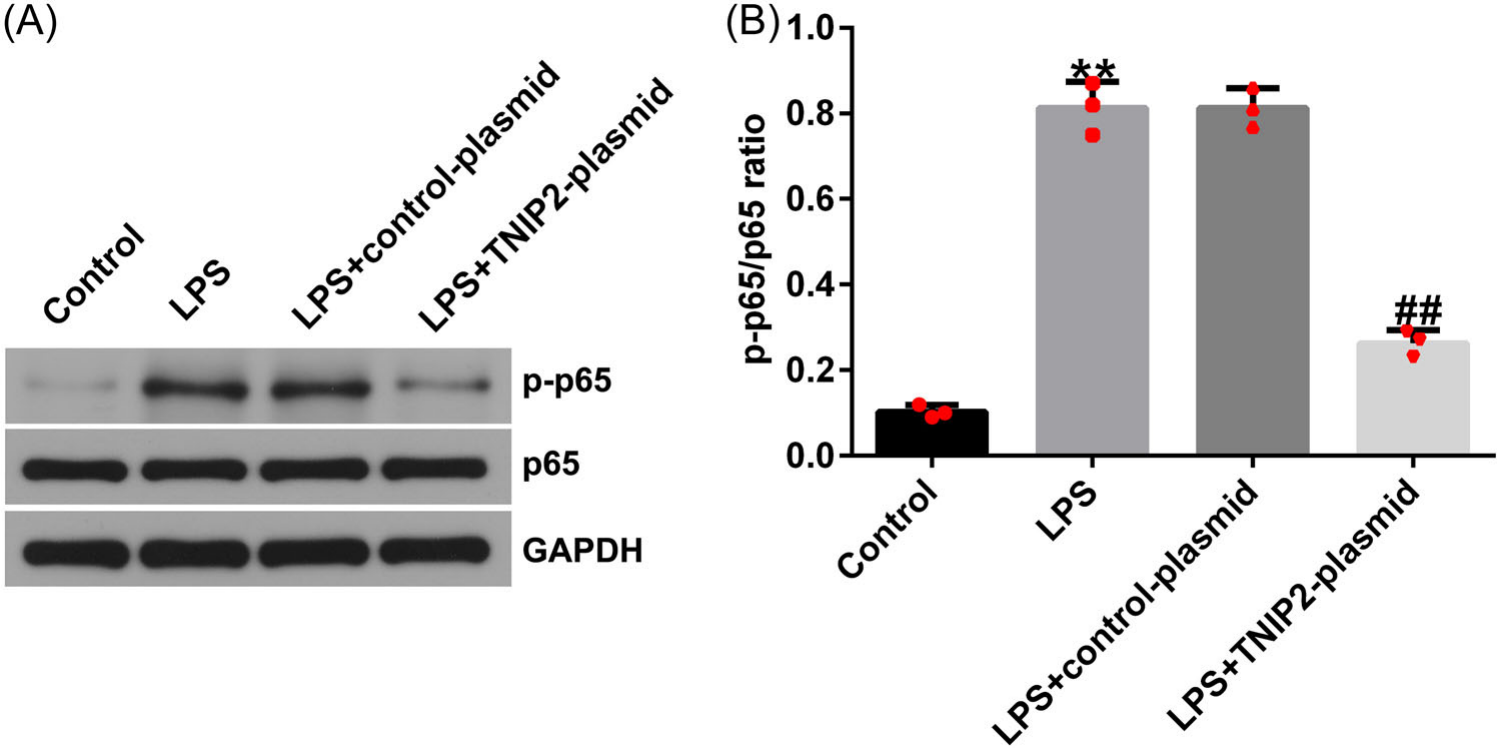
NF‐κB pathway in LPS‐induced hEECs. (A) The p‐p65 and p65 levels were estimated by western blot analysis. (B) The ratio of p‐p65/p65. Data are exhibited as average ± SD of triple single experiments. *N* = 3. **p < .01 versus Control; ^##^ *p* < .01 versus LPS + control‐plasmid. hEECs, human endometrial epithelial cells; LPS, lipopolysaccharide; NF‐κB, nuclear factor‐kappa; SD, standard deviation.

To explore whether NF‐κB was involved in the endometritis pathogenesis, hEECs were pretransfected with control‐plasmid or TNIP2‐plasmid, after 24 h, cells were induced with 1 μg/mL LPS for 3 h. p‐p65 and p65 protein expression was detected by western blot analysis and the p‐p65/p65 ratio was calculated. Compared to the control group, the p‐p65 protein expression and p‐p65/p65 ratio in hEECs in the LPS group were increased, suggesting the activation of NF‐κB pathway by LPS in hEECs. And co‐transfection with the TNIP2‐plasmid reversed this phenomenon (Figure 4A and B).

### 3.5 Protective effect of TNIP2 in endometritis was destroyed by NF‐κB activation

**Figure 5:**
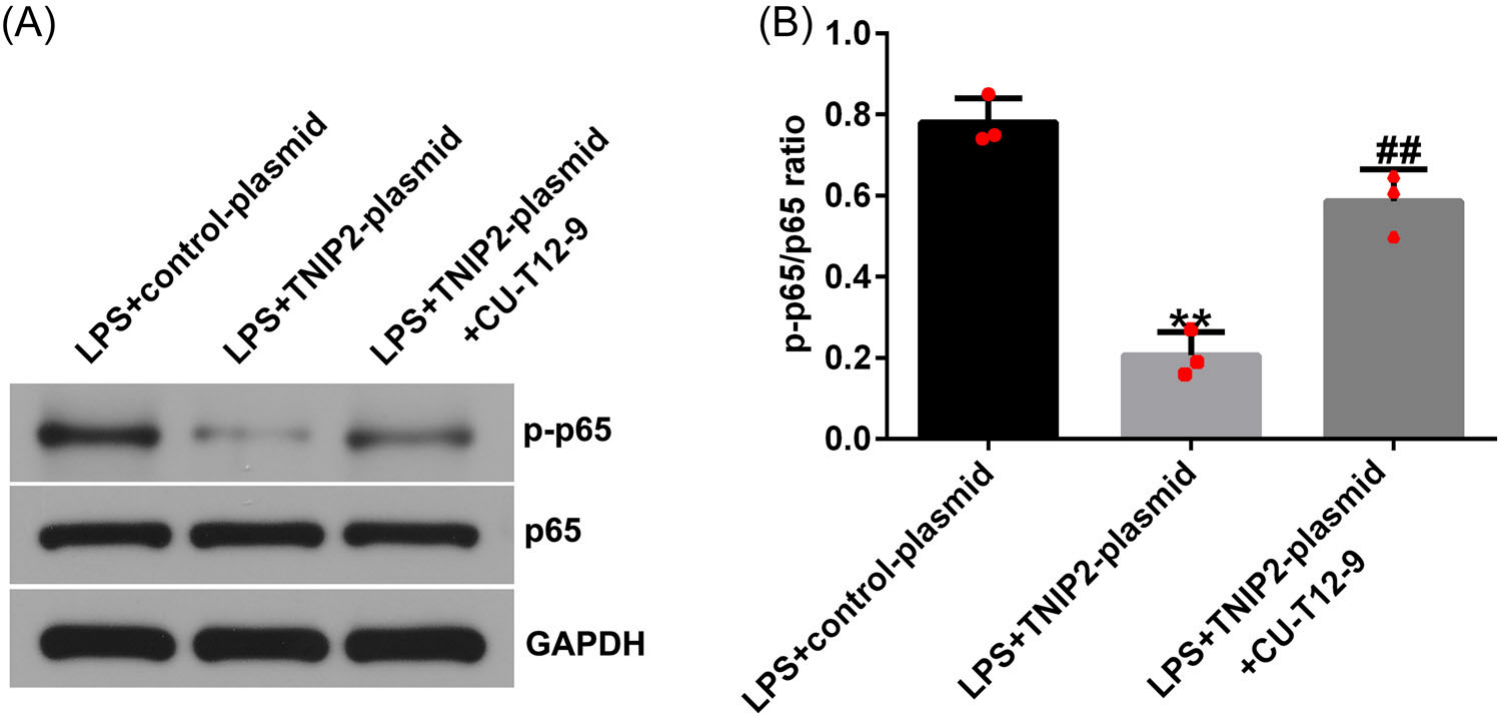
NF‐κB agonist CU‐T12‐9 activated NF‐kB pathway in LPS‐induced hEECs. (A) The p‐p65 and p65 levels were estimated by western blot analysis. (B) The ratio of p‐p65/p65. Data are exhibited as average ± SD of triple single experiments. *N* = 3. **p < .01 versus LPS + control‐plasmid; ^##^ *p* < .01 versus LPS + TNIP2‐plasmid. hEECs, human endometrial epithelial cells; LPS, lipopolysaccharide; NF‐κB, nuclear factor‐kappa; SD, standard deviation; TNIP2, TNFAIP3 interacting protein 2.

**Figure 6:**
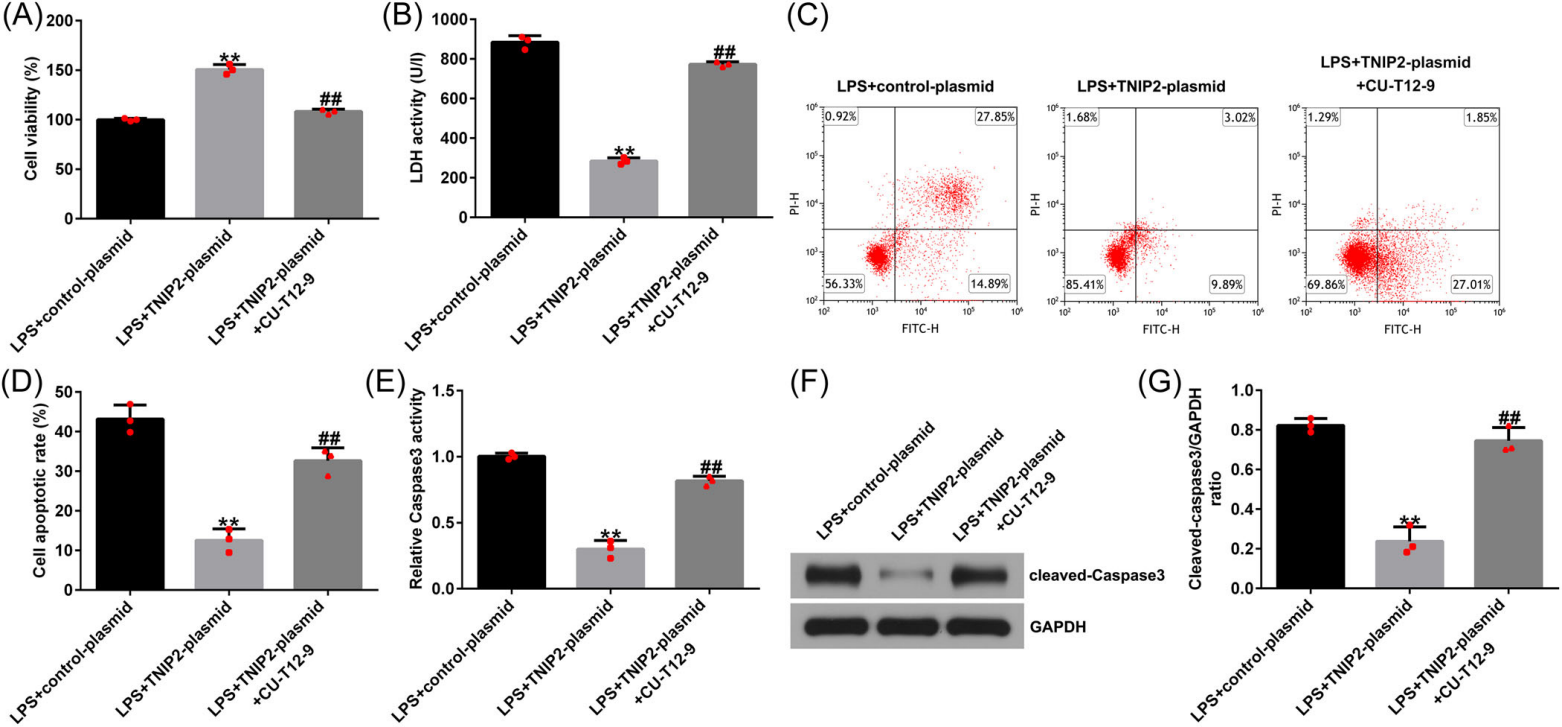
NF‐κB agonist CU‐T12‐9 reversed TNIP2‐induced apoptosis reduction in LPS‐induced hEECs. (A) CCK‐8 was presented to obtain cell viability. (B) The LDH detection kit was carried out to measure LDH activity. (C and D) The apoptosis rate in hEECs was confirmed by FCMs assay. (E) The capase3 activity detection kit was used to measure caspase3 activity in hEECs. (F) The cleaved‐caspase3 protein expression was verified by western blot assay. (G) Cleaved‐caspase3/GAPDH ratio. Data are exhibited as average ± SD of triple single experiments. *N* = 3. **p < .01 versus LPS + control‐plasmid; ^##^ *p* < .01 versus LPS + TNIP2‐plasmid. CCK‐8, cell counting kit‐8; FCM, flow cytometry; hEECs, human endometrial epithelial cells; LDH, lactate dehydrogenase; LPS, lipopolysaccharide; NF‐κB, nuclear factor‐kappa; SD, standard deviation; TNIP2, TNFAIP3 interacting protein 2.

To explore the specific mechanism of TNIP2 in the course of endometritis in this study, hEECs were pretransfected with a control‐plasmid/TNIP2‐plasmid or treated with NF‐κB agonist CU‐T12‐9 for 24 h and then treated with 1 μg/mL LPS for 3 h. In LPS‐induced hEECs, TNIP2‐reduced p‐p65 protein expression and the p‐p65/p65 ratio decline were significantly reversed by CU‐T12‐9 (Figure 5A and B). In addition, the findings indicated that compared with the LPS + control‐plasmid group, TNIP2‐plasmid transfection significantly increased cell viability (Figure 6A), downregulated the LDH activity (Figure 6B), reduced the ratio of apoptosis (Figure 6C and D), and decreased caspase3 activity and the protein pattern of cleaved‐caspase3 (Figure 6E–G) in LPS‐induced hEECs. All of the results described above were reversed by the CU‐T12‐9 treatment.

**Figure 7:**
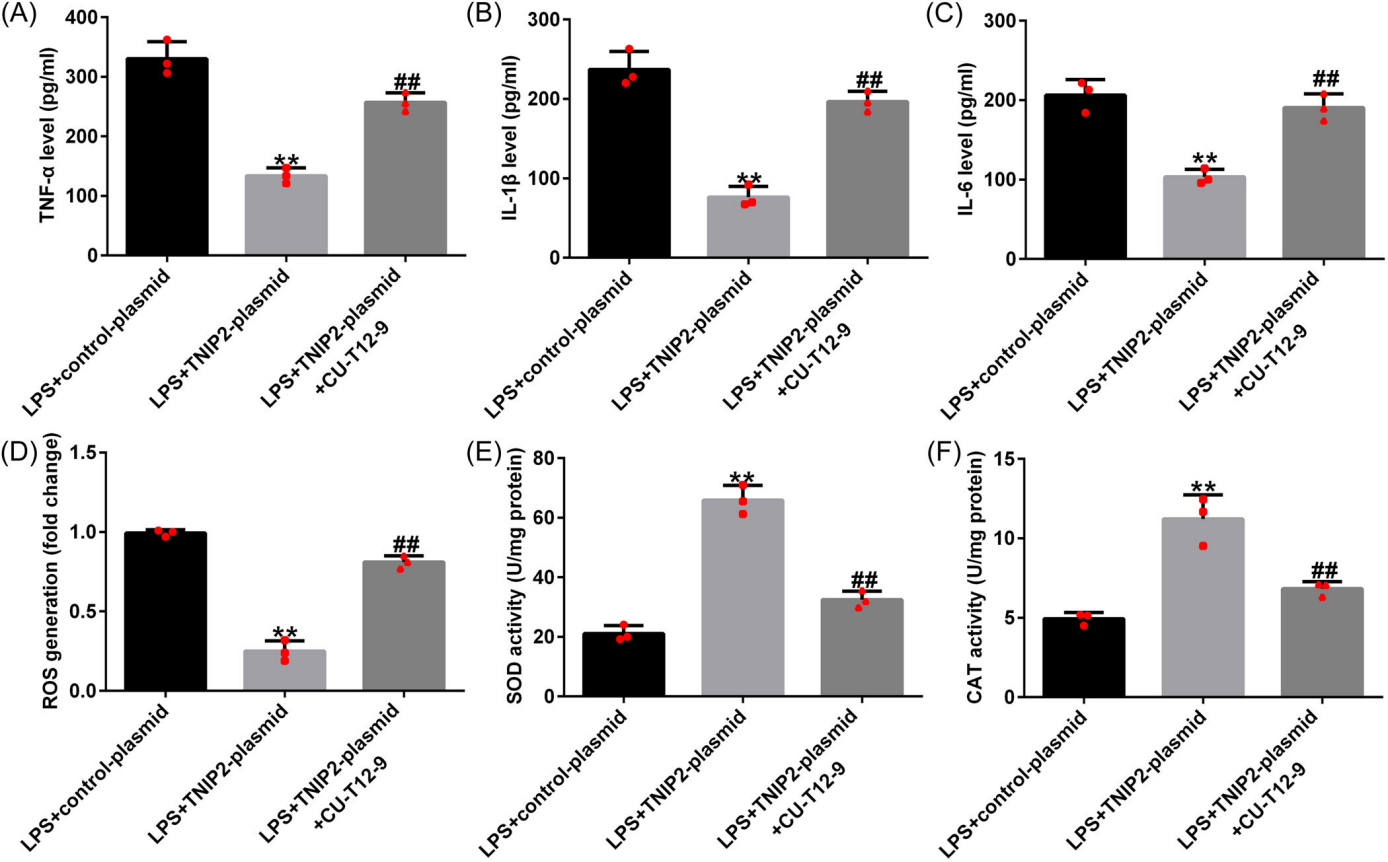
NF‐κB agonist CU‐T12‐9 reversed the TNIP2‐induced inflammation and oxidative stress suppression in LPS‐induced hEECs. (A–C) The concentration of TNF‐α, IL‐1β, and IL‐6 was detected by ELIZA. (D) The ROS kit was used to confirm the ROS release. (E and F) SOD and CAT activity kits were enacted to detect the activity of SOD and CAT. Data are exhibited as average ± SD of triple single experiments. *N* = 3. **p < .01 versus LPS + control‐plasmid; ^##^ *p* < .01 versus LPS + TNIP2‐plasmid. CAT, catalase; ELIZA, enzyme‐linked‐immunosorbent analysis; hEECs, human endometrial epithelial cells; IL, Interleukin; LPS, lipopolysaccharide; NF‐κB, nuclear factor‐kappa; ROS, reactive oxygen species; SD, standard deviation; SOD, superoxide dismutase; TNF‐α, tumor necrosis factor‐alpha; TNIP2, TNFAIP3 interacting protein 2.

Moreover, the TNIP2‐plasmid‐induced TNF‐α, IL‐1β, and IL‐6 secretion downregulation in LPS‐induced hEECs were significantly inhibited by CU‐T12‐9 co‐treatment (Figure 7A–C). Meanwhile, the TNIP2‐plasmid induced reduction in ROS production and the increase in SOD and CAT activity was reversed by CU‐T12‐9 (Figure 7D–F).

## 4 DISCUSSION

Endometritis is an inflammatory disease affecting the female reproductive system.^27^ If inflammation is not controlled in a timely and effective manner, pathogenic microorganisms can invade the endometrium, resulting in repeated episodes of disease and damage to the patient's reproductive system.^28^ Antibiotics are the main clinical treatment; however, they cause many adverse reactions, drug resistance occurs easily, and the disease recurrence rate is high.^29^ Therefore, there is an urgent need to elucidate the latent mechanisms of endometritis and discover efficient therapeutic targets for clinical application.

Recent studies revealed that TNIP2 is involved in several disease processes. Voelkl et al. reported that TNIP2 is involved in cardiovascular diseases.^30^ Klintman et al. have shown that TNIP2 is associated with chronic lymphocytic leukemia.^31^ Yan et al. reported that neuronal damage is alleviated by TNIP2 overexpression.^32^ Furthermore, previous work showed that TNIP2 was involved in Lupus nephriti development.^33^ However, only a few studies have investigated the effects of TNIP2 on endometritis. In this study, effects of TNIP2 on LPS‐induced endometritis were investigated.

Inflammation and oxidative stress are enhanced during endometritis, and endometritis can be alleviated by the inhibition of inflammation and oxidative stress.^34^, ^35^ LPS‐induced inflammation can lead to uncontrolled production of inflammatory mediators such as chemokines, cytokines, and ROS.^36^ ROS could cause oxidative injury to the cells, thus causing the reduction of the activity of CAT and SOD.^37^ In our study, we found that TNIP2 overexpression prevented LPS‐induced endometritis in vitro, evidenced by reduced cell apoptosis, inflammatory response, and inhibited oxidative stress in LPS‐induced hEECs.

NF‐κB signaling is a critical cytoprotective way. There is growing evidence that the NF‐κB signal pathway plays a critical role in human disease. Xu et al. revealed that Polyene Phosphatidylcholine prevented synovial inflammation via inactivation of NF‐κB signaling.^38^ Ma et al. demonstrated that NF‐κBp65 was involved in the mechanism of Jinzhen on novel SARS‐Cov‐2.^39^ Yang et al. demonstrated that the NF‐κB pathway was related to Thioredoxin‐1 mediates neuroprotection of Schisanhenol in SH‐SY5Y cells.^40^ Lin et al. found that loss of lncRNA HCG15 from exosomal prevents acute myocardial ischemic injury via the NF‐kB.^41^ In our research, we found that TNIP2 overexpression notably inhibited the LPS‐induced endometritis via the NF‐κB pathway.

There were also some limitations of this study. First, in this study, experiments were only done in a single in vitro model with a cell line and without the presence of immune cells. Besides, there are significant differences between in vitro models and human diseases themselves, and this was a limitation of this study. Moreover, this study did not explore the role of TNIP2 in the animal model of endometritis.

In conclusion, we described in detail that TNIP2 mitigates LPS‐induced endometritis in vitro. Therefore, our data suggest that TNIP2 may serve as a new target for treating endometritis.

## AUTHOR CONTRIBUTIONS

**Xinxin Qian**: Conceptualization; formal analysis; investigation; writing—original draft; writing—review and editing. **Yan Wang**: Investigation; methodology; software. **Xingmei Li**: Investigation; methodology; software. **Yuewen Li**: Investigation; methodology; software. **Liping Li**: Investigation; methodology; software.

## CONFLICT OF INTEREST STATEMENT

The authors declare no conflict of interest.

## Data Availability

The data sets used and/or analyzed during the current study are available from the corresponding author on reasonable request.
